# Effect of electroacupuncture on the degradation of collagen in pelvic floor supporting tissue of stress urinary incontinence rats

**DOI:** 10.1007/s00192-022-05106-8

**Published:** 2022-02-28

**Authors:** Chaonan Li, Mengyi Yang, Zhiyu Qu, Shuoquan Ruan, Bingli Chen, Jinchuan Ran, Wen Shu, Yuelai Chen, Wenguang Hou

**Affiliations:** Yueyang Hospital of Integrated Traditional Chinese and Western Medicine, Shanghai University of Traditional Chinese Medicine, No.110, Ganhe Road, Shanghai, 200437 China

**Keywords:** Electroacupuncture, Stress urinary incontinence, Collagen, Matrix metalloproteinase, Urodynamics

## Abstract

**Introduction and hypothesis:**

To examine the changes induced by electroacupuncture in stress urinary incontinence (SUI) rats, including the urodynamics and collagen degradation-related cytokine molecular biological expression changes, and to explore the effect and mechanism of EA treatment in SUI.

**Methods:**

Female SPF Sprague-Dawley rats were randomly assigned to five groups (*n* = 10): sham, model, electroacupuncture control, electroacupuncture, and blocker. The leak point pressure (LPP) and maximum bladder capacity (MBC) were measured for each group of rats, and collagen I, collagen III, matrix metalloproteinases (MMPs), and tissue inhibitor of metalloproteinase (TIMPs) in the anterior vaginal wall of rats in each group were determined by reverse transcription-polymerase chain reaction and western blotting. The data were analyzed by one-way analysis of variance or Kruskal-Wallis test.

**Results:**

Electroacupuncture Shenshu (BL23) and Huiyang (BL35) increased the LPP and MBC in SUI rats (*P* < 0.05). Electroacupuncture treatment significantly increased the protein expression of collagen I and collagen III in the anterior vaginal wall of SUI rats (*P* < 0.05) and significantly reduced the protein expression of MMP1, MMP2, and MMP9 (*P* < 0.05).

**Conclusions:**

Electroacupuncture stimulation can alleviate the signs of SUI, and its mechanism is related to the degradation of collagen in the anterior vaginal wall.

**Supplementary Information:**

The online version contains supplementary material available at 10.1007/s00192-022-05106-8.

## Introduction

Stress urinary incontinence (SUI) is defined by the International Continence Society (ICS) as involuntary leakage of the urethral opening during hard or physical activity or during sneezing or coughing [1]. Vaginal delivery is one of the main risk factors for SUI, which can affect the pelvic muscles and nerve and connective tissues, resulting in an insufficient bladder neck and urethral support [2]. The “hammock hypothesis” describes that the anterior vaginal wall and the pelvic fascia together form the supporting structure of the urethra. These structures are attached to the pelvic fascia and tendon arch and combine with the levator ani muscle to form “hammocks.” The urethra is compressed, which can prevent the involuntary loss of urine, when the abdominal pressure increases. The urethral support system is composed of the external structure of the urethra, including the anterior vaginal wall, pelvic fascia, pelvic fascia arch, and levator ani muscle. The normal function of the urethral support system is a necessary condition to promote the transmission of increased abdominal pressure to the urethra [3–5]. Obesity, chronic cough, childbirth, menopausal status, and other factors can cause damage and destruction of the urethral support structure, thereby resulting in involuntary urine leakage [6, 7].

The lack of estrogen after menopause changes the metabolism of connective tissue and reduces the production of collagen, which may lead to the occurrence of SUI [8]. Studies have shown that estrogen receptors are located in the vagina, urethra, bladder, and pelvic floor muscle tissue. The lack of estrogen after menopause is related to the thinning of the submucosa and the atrophic changes in the pelvic floor myofascial structure. Atrophic changes in the structure lead to a decrease in the closure pressure of the urethra, which in turn promotes the emergence of SUI [9, 10]. The occurrence of SUI may be related to an increase in the amount of matrix metalloproteinase (MMP) degraded collagen in the extracellular matrix of the pelvic floor support tissues, resulting in insufficient bladder neck and urethral support. Doxycycline hydrochloride, a matrix metalloproteinase inhibitor, can inhibit the activity of matrix metalloproteinase and reduce the degradation of collagen in pelvic floor supporting tissue [11, 12] to treat SUI.

Clinical studies have shown that electroacupuncture (EA) at lumbosacral points can reduce the amount of urine leakage in SUI patients and can effectively and safely alleviate the symptoms of urinary incontinence and improve the quality of life of SUI patients. After EA treatment, its therapeutic effect will last for some time and can reduce the frequency of SUI [13, 14]. EA treatment can downregulate the expression of matrix metalloproteinase-9 (MMP9), increase the content of collagen in rat tendons that heal, and change the ultrastructure of collagen [15–17]. However, there are few studies on the effect of EA treatment on collagen metabolism in the pelvic floor supporting tissues of SUI rats. We hypothesized that the degradation of extracellular matrix collagen in the anterior vaginal wall of SUI rats increased, and electroacupuncture could reverse this change. In this study, Sprague-Dawley (SD) rats were used to establish an SUI model. The efficacy of EA in the treatment of SUI was observed through urodynamic measurements, and whether there was a relationship between collagen degradation in the tissues that support the rat pelvic floor and EA treatment of SUI was explored.

## Materials and methods

### Animals and study design

The experimental animals were female SPF Sprague-Dawley rats, all provided by Beijing Vital River Laboratory Animal Technology Co., Ltd. They weighed 230 ± 20 g. During the experiment, the animals were kept in the Experimental Animal Center of Shanghai University of Traditional Chinese Medicine in a quiet and clean environment, with a 12-h:12-h day and night light cycle, 4–5 rats in each cage, ad libitum access to food and water, an indoor constant temperature of 22 ± 2 °C, and humidity of 50%–55%. All rats were allowed to adapt to the breeding environment for 1 week before the experiment. All animal experiments carried out in the study were approved by the animal research ethics committee of Shanghai University of Traditional Chinese Medicine (no. PZSHUTCM 191018008).

In this study, rats were randomly assigned to five groups (*n* = 10): sham, model, electroacupuncture control (EAC), EA, and blocker. Except for sham rats, all rats underwent vaginal balloon dilatation and bilateral oophorectomy to replicate the SUI model [18]. Briefly, after anesthesia (sodium pentobarbital, 30 mg/kg, i.p.), an 8-Fr latex Foley catheter was inserted into the rat vagina, and 4.0 ml of sterile saline was slowly pushed into the balloon to expand the vagina. The catheter was sutured and fixed, the rat pubic symphysis was placed on the edge of the rat model frame so that the urinary catheter drooped, and a 120-g weight was suspended in the drooping injection port. The catheter was removed after 4 h. After regular feeding for 1 week, the rats were anesthetized. Then, both ovaries were removed by midline abdominal incision, and urodynamics were measured after regular feeding for 2 weeks.

### Urodynamic determination

In the experiment, there were two urodynamic measurements (to reduce the discomfort of the rats, the measurements were made after anesthesia): 1 week after modeling and 1 day after the end of treatment. After emptying the bladder, a 0.7-mm epidural catheter was inserted 2 cm into the rat bladder before fixing it. One end was connected to a microinjection pump through a three-way connector to the bladder, and sterile saline was injected at a rate of 0.3 ml/min. One end was connected to a urodynamic testing instrument (Bonito urodynamic detector, Laborie Medical Technology Company of Canada). The urodynamic testing instrument was calibrated to “0,” and the test was started. When the first drop of fluid appeared at the urethral orifice, perfusion was stopped. At this time, the bladder capacity (perfusion time multiplied by perfusion speed) and intravesical pressures were recorded to determine the leak point pressure (LPP) and maximum bladder capacity (MBC). The sneeze test was carried out after the first urodynamic measurement [19]. The successful model rats (*n* = 32) were randomly assigned to four groups: model (*n* = 8), EAC (n = 8), EA (n = 8), and blocker (*n* = 8). Within 24 h after the end of the treatment, the rats were killed under anesthesia with 3% sodium pentobarbital (0.5 ml/100 g). The abdominal cavity was opened along the median incision of the lower abdomen, the bladder was taken as the sign, the tissue around the bladder was cut with ophthalmic scissors, the urethra was separated from the outside, and the tissue was adhered to the urethra; that is, the anterior wall of the vagina and the anterior vaginal wall were removed, immediately frozen in liquid nitrogen, and stored at −80 °C until use. Some animals in each group (*n* = 4) were only used for RT-PCR determination. Other animals (*n* = 4) were only used for western blotting determination.

### Treatment protocol

The EA group was treated by electrical stimulation (density wave, 4/20 Hz, 20 min) at the acupoints of Shenshu (BL23, on both sides of the spinous process on the lower back) and Huiyang (BL35, positioned almost vertically underneath the periosteum approximately 5 mm lateral to the midline of the coccyx, with bilateral symmetry), determined relative to their anatomical locations described in the WHO guidelines for human acupoints [20]. A disposable acupuncture needle (diameter, 0.25 mm) was connected to an SDZ-V EA instrument (Shanghai, China). EA treatment was performed once per day and continued for 3 days. For the EAC group, stimulation was at the Shenshu control point (1 cm beside Shenshu) and at the Huiyang control point (1 cm beside Huiyang). The stimulation settings were the same as in the EA group. The blocker group was given doxycycline hydrochloride at 30 mg/kg/d diluted with normal saline and then administered intragastrically for 3 consecutive days [21].

### Quantitative real-time polymerase chain reaction

Total RNA was extracted with TRIzol reagent (Invitrogen, Carlsbad, CA), and the Agilent 2100 chip system was used to evaluate the quality of each RNA sample. The RNA concentration of each sample was measured with a Nanodrop spectrophotometer (Nanodrop Technologies, Wilmington, DE). Quantitative analysis was performed using an Agilent 2100 chip system (Agilent Technologies, Palo Alto, CA). The reverse transcription reaction was initiated with oligo (dT), glyceraldehyde 3-phosphate dehydrogenase (Gapdh) was used as an internal control, and qPCR was performed in duplicate. See Table 1 for primer sequences. The cycling conditions were 10 min at 95 °C, then 40 cycles of 15 s at 95 °C and 1 min at 60 °C. After the cycle, the melting operation was performed at 95 °C for 15 s, 60 °C for 1 min, and 95 °C for 15 s to control the specificity of the product. Using gapdh as the internal reference gene, the CT value of each sample was corrected, where each group of samples had three replicates for each gene. The fold change of the target gene cDNA relative to Gapdh was determined as follows: fold change = 2^−ΔΔCt^, fold change = (Ct_Target_-Ct_Gapdh_) test - (CT_Target_-Ct_Gapdh_) control. The Ct values were defined as the number of PCR cycles at which the fluorescence signals were detected.

**Table 1:** Sequences of primers for real-time PCR

Symbol	Forward primer	Reverse primer
Gapdh	TGACTTCAACAGCGACACCCA	CACCCTGTTGCTGTAGCCAAA
Collagen	TGCTGCCTTTTCTGTTCCTT	AAGGTGCTGGGTAGGGAAGT
Collagen II	GTCCACGAGGTGACAAAGGT	CATCTTTTCCAGGAGGTCCA
MMP1	GCATGCTTAGCCTTCCTTTG	CTGAAACACGGGGAAACTGT
MMP2	AGCTCCCGGAAAAGATTGAT	TCCAGTTAAAGGCAGCGTCT
MMP9	CACTGTAACTGGGGGCAACT	AGAGTACTGCTTGCCCAGGA
TIMP1	TCCCCAGAAATCATCGAGAC	TCAGATTATGCCAGGGAACC

### Western blotting

The tissue was homogenized in cold lysis buffer (Beyotime Biotechnology Co., Haimen, Jiangsu, China) and centrifuged at 13,200 rpm for 15 min at 4 °C. The supernatant containing total protein was quantified by an Enhanced BCA Protein Assay Kit (Beyotime Biotechnology Co.). The samples were separated on a 10% sodium dodecyl sulfate-polyacrylamide gel electrophoresis gel (loading 30 μg of total protein in each lane) and were transferred to a polyvinylidene fluoride membrane (Millipore, Bedford, MA). The membrane was blocked with 5% skimmed milk overnight at 4 °C and then incubated with primary antibodies recognizing Gapdh (mouse monoclonal, 1:1000; BioTNT), collagen I(rabbit polyclonal, 1:1000; Novus), collagen III (mouse monoclonal, 1:1000; Abcam), MMP1 (rabbit polyclonal, 1:500; Invitrogen), MMP2(mouse monoclonal, 1:1000; Abcam), MMP9(rabbit monoclonal, 1:1000; Abcam), and TIMP1(mouse polyclonal, 1:200; Invitrogen). The primary antibodies were incubated together at 22 °C for 2 h. Then, the membrane was incubated with horseradish peroxidase-conjugated anti-mouse (1:5000; Abcam) or goat anti-rabbit (1:5000; Abcam) secondary antibodies.

### Statistical analysis

The data were compiled using Excel 2019 software, and SPSS 25.0 was used for statistical analysis. The measurement data that satisfied the normal distribution were analyzed by one-way analysis of variance to judge the differences between groups according to the data type. Multiple comparisons were performed with the LSD method, and the data are expressed as the mean ± SD. The measurement data that did not meet the normal distribution or met the normal distribution but had variance that was not uniform were analyzed by the Kruskal-Wallis test. Multiple comparisons were performed by Kruskal-Wallis one-way ANOVA (k samples), and the data are presented as the M (P25, P75), with values of *P* < 0.05 considered significant.

## Results

### EA improves the LPP and MBC in rats with SUI

**Fig. 1:**
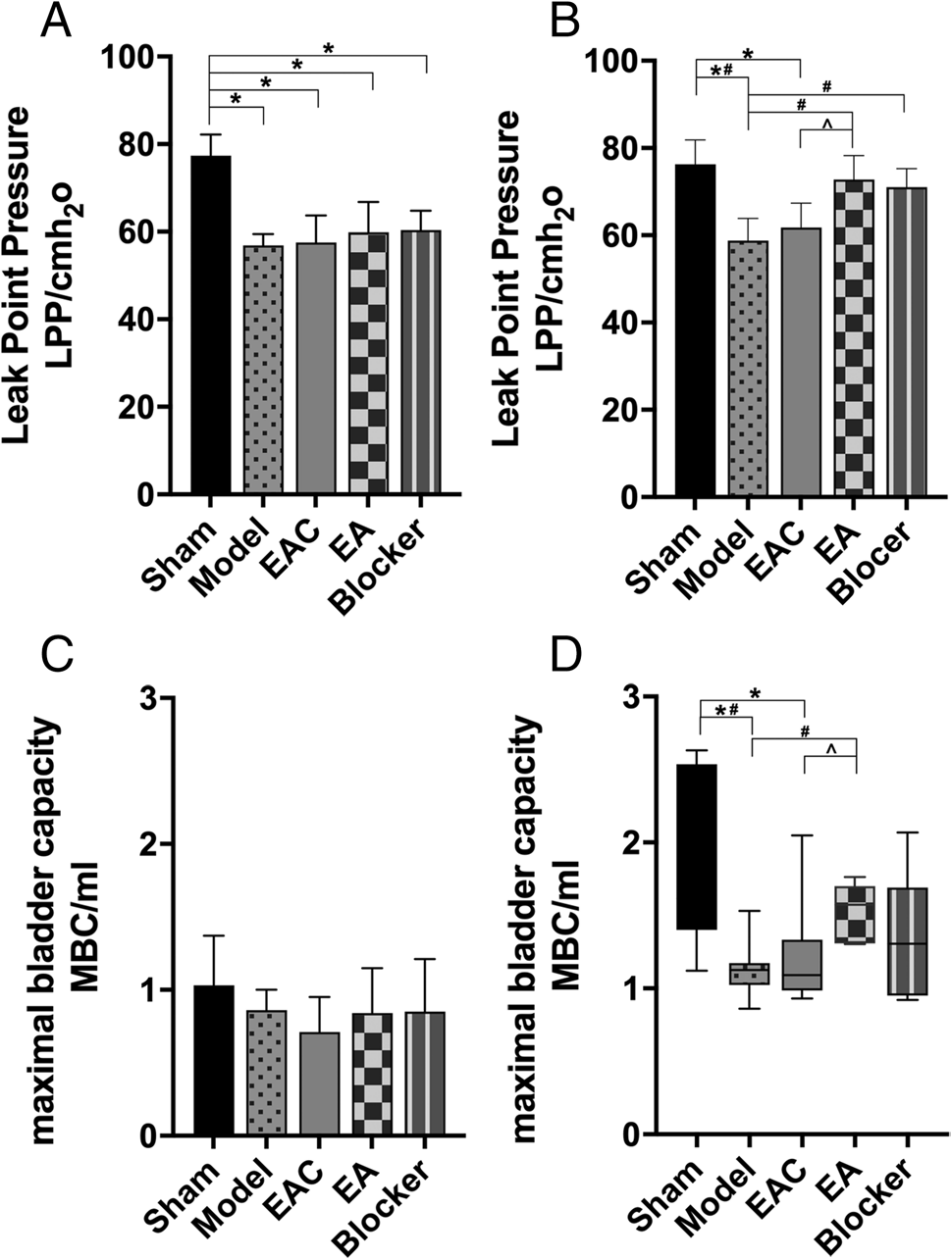
EA improves the LPP (**A**, **B**) and MBC (**C**, **D**) in rats with SUI. The effects of EA on rats with SUI (sham = 8, model = 8, EAC = 8, EA = 8, blocker = 8). **A** The measurement of LPP before treatment was analyzed via one-way analysis of variance (ANOVA) (F _(4,35)_ = 21.103, *P* = 0.001, <0.05). **B** The measurement of LPP after treatment was analyzed via ANOVA (F _(4,35)_ = 16.280, *P* = 0.001, < 0.05). **C** The measurement of MBC before treatment was analyzed via ANOVA (F _(4,35)_ = 1.210, *P* = 0.324, > 0.05). (**D**) The measurement of LPP after treatment was analyzed via the Kruskal-Wallis test (H = 12.938, *P* = 0.012, < 0.05). ^*^P < 0.05 compared with the sham group; ^#^*P* < 0.05 compared with the model group

A comparison of the LPP of rats in each group before treatment is shown in Fig. 1A. After vaginal dilatation and bilateral oophorectomy in rats, the bladder LPP of rats in each group after modeling was significantly lower than that of the sham group(*P* < 0.05). A comparison of the LPP of rats in each group after treatment is shown in Fig. 1B. The results showed that electroacupuncture and blocker stimulation could significantly increase the LPP of SUI rats, while electroacupuncture control group could not reverse the LPP of SUI rats. A comparison of the MBC of rats in each group before treatment is shown in Fig. 1C. There was no statistically significant difference in the MBC of the five groups of rats (*P* = 0.324, > 0.05), indicating that sham (1.03 ± 0.34) rats and model (0.86 ± 0.14), EAC (0.71 ± 0.24), EA (0.84 ± 0.30), and blocker (0.85 ± 0.36) rats had no difference in the MBC. A comparison of the MBC of rats in each group after treatment is shown in Fig. 1D. Electroacupuncture stimulation can significantly improve the MBC of SUI rats, while electroacupuncture control group cannot reverse the MBC of SUI rats.

### EA slows the degradation of collagen in the anterior vaginal wall of SUI rats

**Fig. 2:**
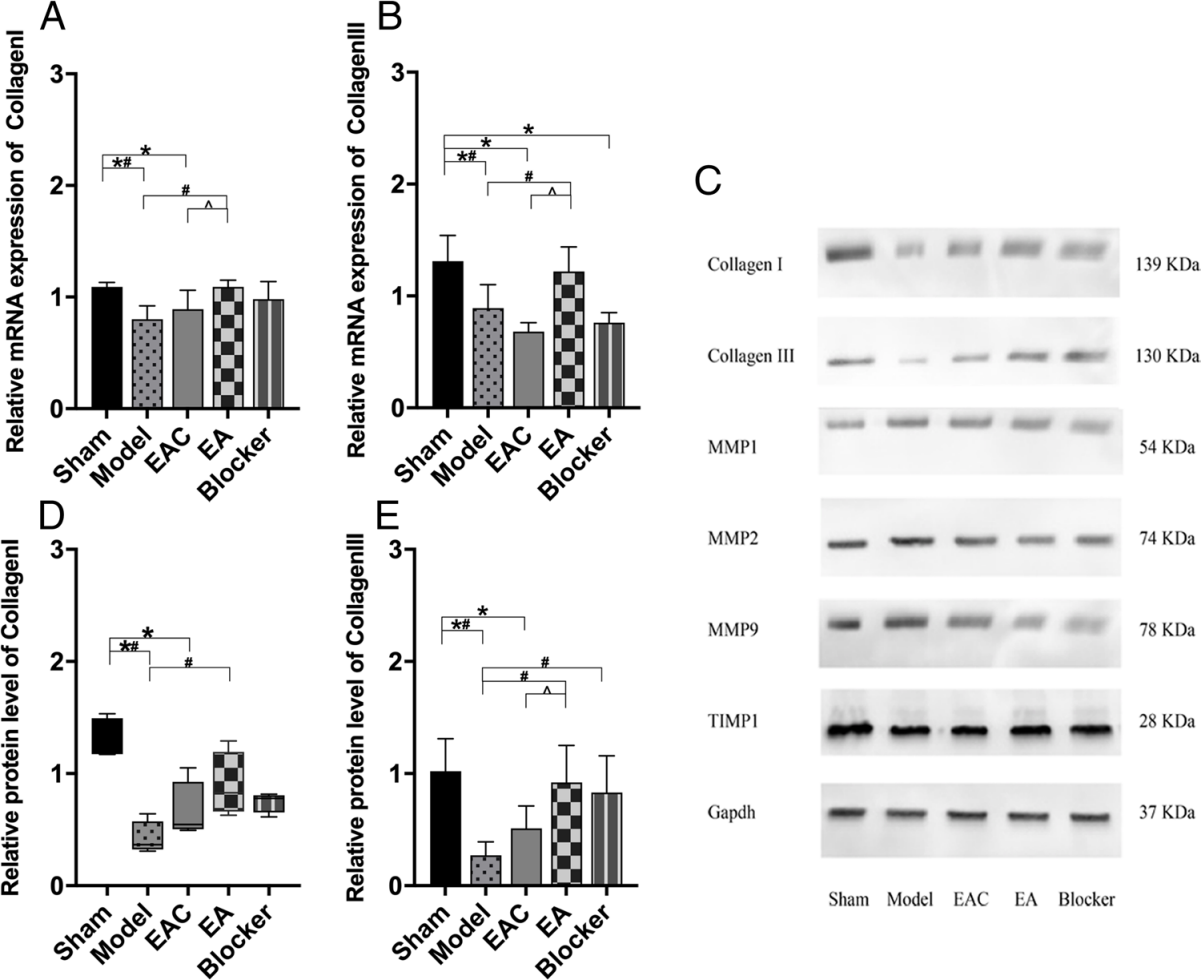
The mRNA and protein expression of collagen I, collagen III in the anterior vagina of each group (sham = 4, model = 4, EAC = 4, EA = 4, blocker = 4). **A** Collagen I mRNA expression in each group was analyzed via ANOVA (F _(4,15)_ = 4.189, *P* = 0.018, < 0.05). **B** Collagen III mRNA expression in each group was analyzed via ANOVA (F _(4,15)_ = 10.003, *P* = 0.001, < 0.05). **C** The protein expression bands of collagen I, collagen III, MMP1, MMP2, MMP9, and TIMP1 in the anterior vagina of each group. **D** Collagen I protein expression in each group was analyzed via the Kruskal-Wallis test (H = 13.129, *P* = 0.011, < 0.05). **E** Collagen III protein expression in each group was analyzed via ANOVA (F _(4,15)_ = 5.484, *P* = 0.006, < 0.05). ^*^P < 0.05 compared with the sham group; ^#^*P* < 0.05 compared with the model group. ^^^*P* < 0.05 compared with the EAC group

The mRNA expression of collagen I and collagen III in the anterior vaginal wall of rats in each group is shown in Fig. 2A and B. In the model group, vaginal dilatation and bilateral oophorectomy significantly decreased the mRNA expression levels of collagen I and collagen III in in anterior vaginal wall compared with the sham group (*P* < 0.05 each). This decrease was mitigated by EA treatment (each *P* < 0.05, EA versus model). The protein expression levels of collagen I, collagen III, MMP1, MMP2, MMP9, and TIMP1 in the anterior vaginal wall of rats in each group are shown in Fig. 2C. Similarly, the protein levels of collagen I and collagen III in the anterior vaginal wall were both decreased in the model group compared with the sham group (each *P* < 0.05) (Fig. 2D and E). EA treatment significantly mitigated the decrease in collagen I and collagen III protein expression induced by vaginal dilatation and bilateral oophorectomy(each *P* < 0.05). However, for the rats in the EAC group, the levels of collagen I and collagen III protein and mRNA did not change significantly compared with the model group (each *P* > 0.05).

**Fig. 3:**
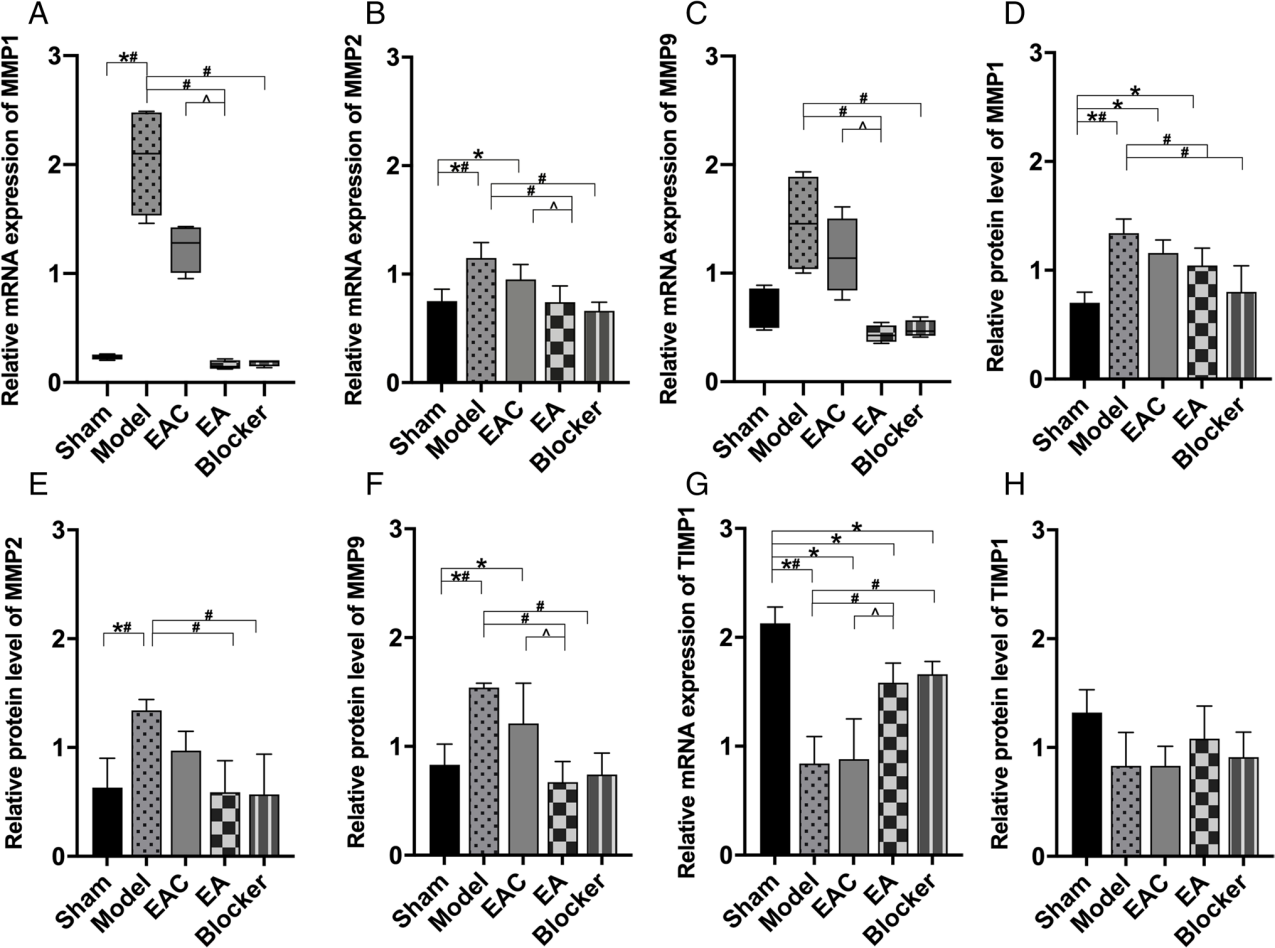
mRNA and protein expression of MMP1, MMP2, MMP9, and TIMP1 in the anterior vagina of each group (sham = 4, model = 4, EAC = 4, EA = 4, blocker = 4). **A** MMP1 mRNA expression in each group was analyzed via the Kruskal-Wallis test (H = 16.857, *P* = 0.002, < 0.05). **B** MMP2 mRNA expression in each group was analyzed via ANOVA (F _(4,15)_ = 9 .729, *P* = 0.001, < 0.05). **C** MMP9 mRNA expression in each group was analyzed via the Kruskal-Wallis test (H = 15.200, *P* = 0.002, < 0.05). **D** MMP1 protein expression in each group was analyzed via ANOVA (F _(4,15)_ = 10.588, *P* = 0.001, < 0.05). **E** MMP2 protein expression in each group was analyzed via ANOVA (F _(4,15)_ = 6.555, *P* = 0.003, < 0.05). **F** MMP9 protein expression in each group was analyzed via ANOVA (F _(4,15)_ = 10.634, *P* = 0.001, < 0.05). **G** TIMP1 mRNA expression in each group was analyzed via ANOVA (F _(4,15)_ = 22.675, *P* = 0.001, < 0.05). **H** TIMP1 protein expression in each group was analyzed via ANOVA (F _(4,15)_ = 2.767, *P* = 0.066, > 0.05). ^*^P < 0.05 compared with the sham group; ^#^*P* < 0.05 compared with the model group. ^^^*P* < 0.05 compared with the EAC group

The mRNA expression of MMP1, MMP2, and MMP9 in the anterior vaginal wall of rats in each group is shown in Fig. 3A, B, and C. In the model group, vaginal dilatation and bilateral oophorectomy significantly increased the mRNA expression levels of MMP1 and MMP2 in anterior vaginal wall compared with the sham group (*P* < 0.05 each). This increase was mitigated by EA treatment (each *P* < 0.05, EA versus model). Similarly, the protein levels of MMP1, MMP2 and MMP9 in the anterior vaginal wall were both increased in the model group compared with the sham group (each *P* < 0.05) (Fig. 3D, E, and F). EA treatment significantly mitigated the increase in MMP1, MMP2, and MMP9 protein expression induced by vaginal dilatation and bilateral oophorectomy (each *P* < 0.05). However, in the rats in the EAC group, the levels of MMP1, MMP2, and MMP9 protein and mRNA did not change significantly compared with the model group (each *P* > 0.05). The mRNA expression of TIMP1 in the anterior vaginal wall of rats in each group is shown in Fig. 3G. In the model group, vaginal dilatation and bilateral oophorectomy significantly increased the mRNA expression levels of TIMP1 in anterior vaginal wall compared with the sham group (*P* < 0.05 each). This increase was mitigated by EA treatment (each *P* < 0.05, EA versus model). There was no statistically significant difference in TIMP1 protein expression among the five groups of rats (*P* = 0.066, > 0.05) (Fig. 3H), wherein the expression was similar among the sham (1.32 ± 0.21), model (0.83 ± 0.31), EAC (0.82 ± 0.18), EA (1.08 ± 0.30), and blocker (0.91 ± 0.23) rats.

## Discussion

This study shows that EA treatment can increase the LPP and MBC in SUI rats and reverse the downregulation of collagen I and collagen III in the anterior vaginal wall of SUI rats and the upregulation of MMP1, MMP2, and MMP9. This response is related to the decrease in collagen degradation in the anterior vaginal wall of SUI rats. Previous clinical studies have shown that EA lumbosacral acupoints can reduce urine leakage in SUI patients. EA can effectively and safely alleviate the symptoms of urinary incontinence and improve the quality of life of SUI patients. Current studies have shown that EA treatment can modify the LPP and MBC of SUI rats to improve the signs of SUI.

The occurrence of SUI at the anatomical level is manifested as insufficient support of the bladder neck and urethra. In pelvic floor support tissue, collagen in the extracellular matrix is the main component that provides structural support and mechanical stability for tissues and organs. The collagen content in pelvic floor tissue mainly depends on the balance between MMPs and TIMPs secreted by fibroblasts. When the degradation of collagen by MMPs is enhanced, it reduces the support of pelvic floor tissues, affects the position of the bladder neck and urethra, prevents the urethra from closing, and thus triggers SUI. Collagen is the most abundant protein in the extracellular matrix. It is an insoluble fibrin with a triple-helix conformation, accounting for approximately one-quarter of the total protein mass. Fiber-forming collagen is stabilized by nonreducible covalent cross-links between specific triple-helix domains and telopeptides, such as interstitial collagen (collagen I, collagen II, and collagen III), collagen V, and collagen XI [22]. Its main function is to provide structural support and mechanical stability for tissues and organs. The types of collagen contained in human pelvic tissues are mainly collagen I (related to tissue stiffness) and collagen III (related to tissue elasticity). The collagen content in the pelvic floor tissue mainly depends on the balance between MMPs secreted by fibroblasts and their inhibitory factors. MMPs are involved in processes including embryo formation, normal tissue remodeling, wound healing, and angiogenesis [23]. For example, the MMP1, MMP8, and MMP13 collagenases can cut collagen I, collagen II, and collagen III into 3/4 and 1/4 fragments according to specific sites, while MMP2, which is a gelatinase, can cut the abovementioned fragments into amino acids [24, 25]. Organisms contain endogenous TIMPs, which form complexes by interacting with Zn^2+^ in unactivated MMPs and inhibiting the activity of MMPs to reduce their degradation of collagen in the extracellular matrix. In this study, we selected doxycycline hydrochloride as a blocker of MMPs as the drug treatment group. Doxycycline hydrochloride is often used as an antibiotic to treat gram-positive or -negative bacterial infections. In addition, a study found that doxycycline hydrochloride can directly bind to Zn^2+^ or Ca^2+^, target the active site of MMPs, inhibit the activation of zymogen, and reduce the activity of MMPs [26, 27].

The deficiency of this study is the limited sample size and the limited ability to detect protein. In the process of collagen metabolism, there may be some other cytokines, such as calpain 2 and integrin β1, and transforming growth factor β1, etc. [28, 29]. These cytokines may have participated in the synthesis of collagen but were not detected in this study. We should also note that we need to conduct a comprehensive study on other proteins and signaling pathways in the extracellular matrix to fully explain the effect of the extracellular matrix on micturition function. With the deepening of our understanding of the pathophysiology of the bladder and urethra, the prevention of stress urinary incontinence is becoming increasingly important. Some studies have shown that the increase in matrix metalloproteinase expression is accompanied by intestinal inflammation, and there is a relationship between the composition of the intestinal microbiome and muscle quality [30, 31]. For example, *Bacteroides fragilis* can not only combine collagen binding protein (CBP1) with type I collagen to encode the gene for adhering collagen [32] but also convert plasminogen in plasma into plasmin through the outer membrane protein bfp60, thereby increasing the degradation of fibrin and collagen [33]. It may be an effective measure to prevent or treat stress urinary incontinence in the future by correcting intestinal ecological imbalance or changing intestinal microbial composition.

In conclusion, the results of this study show that after treatment with EA in SUI rats, the expression of MMP1, MMP2, and MMP9 is downregulated, and the expression of collagen I and collagen III is upregulated. The metabolism of extracellular matrix collagen also involves cytokines related to collagen synthesis. These different factors may have a combined effect on the metabolism of collagen. We found that after 3 days of EA intervention, the damaged tissues of SUI rats have some functional improvements and morphological changes, and longer treatment time may have higher curative effect. We are also conducting research in this regard, which you will see in our future work.

## Supplementary Information

ESM 1 (PNG 281 kb)

## References

[1] D'Ancona C, Haylen B, Oelke M (2019). The International Continence Society (ICS) report on the terminology for adult male lower urinary tract and pelvic floor symptoms and dysfunction. Neurourol Urodyn.

[2] Cavalcanti GA, Manzano GM, Nunes KF (2013). Electrophysiological evaluation of the pudendal nerve and urethral innervation in female stress urinary incontinence. Int Urogynecol J..

[3] DeLancey JOL (1994). Structural support of the urethra as it relates to stress urinary incontinence: the hammock hypothesis. Am J Obstet Gynecol.

[4] de Vries AM, Venema Pieter L, Heesakkers John PFA (2018). Midurethral support is also necessary for reflex closure of the urethra. Neurourol Urodyn.

[5] Ashton-Miller JA, Howard D, Delancey JOL (2001). The functional anatomy of the female pelvic floor and stress continence control system. Scand J Urol Nephrol.

[6] Li T, Zhang YJ, Zhang HL (2019). Prevalence and risk factors of stress urinary incontinence among perimenopausal women and its influence on daily life in women with sexual desire problem. Curr Med Sci.

[7] Wang K, Xu X, Jia G (2020). Risk factors for postpartum stress urinary incontinence: a systematic review and meta-analysis. Reprod Sci..

[8] Frani D, Fistoni I. Laser therapy in the treatment of female urinary incontinence and genitourinary syndrome of menopause: an update. Biomed Res Int. 2019:1–9. 10.1155/2019/1576359. 10.1155/2019/1576359 PMC6582847 31275962

[9] Robinson D, Toozs-Hobson P, Cardozo L (2013). The effect of hormones on the lower urinary tract. Menopause Int.

[10] Blakeman PJ, Hilton P, Bulmer JN (2001). Cellular proliferation in the female lower urinary tract with reference to oestrogen status. BJOG Int J Obstet Gynaecol.

[11] Fujita H, Sakamoto N, Ishimatsu Y (2011). Effects of doxycycline on production of growth factors and matrix metalloproteinases in pulmonary fibrosis. Respiration.

[12] Saimin J, Hendarto H, Soetjipto S (2019). The effect of tomato juice on the expression of matrix metalloproteinase-2 (MMP-2) and type-1 collagen on the vaginal wall of the menopausal rats. Bali Med J.

[13] Liu Z, Liu Y, Xu H (2017). Effect of electroacupuncture on urinary leakage among women with stress urinary incontinence: a randomized clinical trial. JAMA.

[14] Xu Huanfang, Liu Baoyan, Wu Jiani, et al. A pilot randomized placebo controlled trial of electroacupuncture for women with pure stress urinary incontinence. PLoS One. 2016;11(3):e0150821. 10.1371/journal.pone.0150821 PMC4784883 26960195

[15] Lin X, Liping Chen X, Yao. (2015). Effects of electroacupuncture preconditioning with different duration on matrix metalloproteinase-9 and vascular endothelial growth factor of blood-brain barrier in rats with cerebral ischemia-reperfusion. Acupuncture research.

[16] de Almeida, de Aro, Guerra, et al. Electroacupuncture increases the concentration and organization of collagen in a tendon healing model in rats. Connect Tissue Res. 2012;53(6):542–7. 10.3109/03008207.2012.710671 22891942

[17] de Almeida, de Freitas KM, Letícia Prado O, et al. Acupuncture increases the diameter and reorganisation of collagen fibrils during rat tendon healing. Acupuncture Med: J British Med Acupuncture Soc. 2015;33(1):51–7. 10.1136/acupmed-2014-010548 25138672

[18] Resplande J, Gholami SS, Graziottin TM (2002). Long-term effect of ovariectomy and simulated birth trauma on the lower urinary tract of female rats. J Urol.

[19] Kaiho Y, Kamo I, Chancellor MB (2007). Role of noradrenergic pathways in sneeze-induced urethral continence reflex in rats. Am J Physiol Renal Physiol.

[20] WHO. Standard Acupuncture Point Locations in the Western Pacific Region. 2008.

[21] Curci JA, Petrinec D, Liao S (1998). Pharmacologic suppression of experimental abdominal aortic aneurysms: a comparison of doxycycline and four chemically modified tetracyclines. J Vasc Surg.

[22] Lavinia A, Anita L, Donata O. From structure to phenotype: impact of collagen alterations on human health. Int J Mol Sci. 2018;19(5):1407. 10.3390/ijms19051407 PMC5983607 29738498

[23] Visse R, Nagase H (2003). Matrix metalloproteinases and tissue inhibitors of metalloproteinases: structure, function, and biochemistry. Circulat Res: J Am Heart Assoc..

[24] Aimes RT, Quigley JP (1995). Matrix metalloproteinase-2 is an interstitial collagenase: inhibitor-free enzyme catalyzes the cleavage of collagen fibrils and soluble native type I collagen generating the specific ¾- and ¼-length fragments. J Biol Chem.

[25] Patterson ML, Atkinson SJ, Knäuper V (2001). Specific collagenolysis by gelatinase A, MMP-2,is determined by the hemopexin domain and not the fibronectin-like domain. FEBS Lett.

[26] Smith GN, Mickler EA, Hasty KA (1999). Specificity of inhibition of matrix metalloproteinase activity by doxycycline: relationship to structure of the enzyme. Arthritis Rheum.

[27] Hanemaaijer R, Visser H, Koolwijk P (1998). Inhibition of MMP synthesis by doxycycline and chemically modified tetracyclines (CMTs) in human endothelial cells. Adv Dent Res.

[28] Li Y, Liu C, Li B, Hong S, Min J, Hu M, Tang J, Wang T, Yang L, Hong L (2019). Electrical stimulation activates calpain 2 and subsequently upregulates collagens via the integrin β1/TGF-β1 signaling pathway. Cell Signal.

[29] Li Y, Li BS, Liu C (2019). Effect of integrin β1 in the treatment of stress urinary incontinence by electrical stimulation. Mol Med Rep.

[30] Heimesaat MM, Dunay IR, Fuchs D (2011). Selective gelatinase blockage ameliorates acute DSS colitis. Eur J Microbiol Immunol (Bp).

[31] Siddharth J, Chakrabarti A, Pannérec A (2017). Ageing and sarcopenia associate with specific interactions between gut microbes, serum biomarkers and host physiology in rats. Ageing (Albany NY).

[32] Galvão BP, Weber BW, Rafudeen MS (2014). Identification of a collagen type I adhesin of *Bacteroides fragilis*. PLoS One.

[33] Ferreira Ede O, Teixeira FL, Cordeiro F (2013). The Bfp60 surface adhesin is an extracellular matrix and plasminogen protein interacting in *Bacteroides fragilis*. Int J Med Microbiol.

