# Supplementary figures and images for: Effect of electroacupuncture on the degradation of collagen in pelvic floor supporting tissue of stress urinary incontinence rats
Source: Int Urogynecol J. 2022 Feb 28;33(8):2233–40. doi: 10.1007/s00192-022-05106-8 (PMC9343271; doi:10.1007/s00192-022-05106-8)

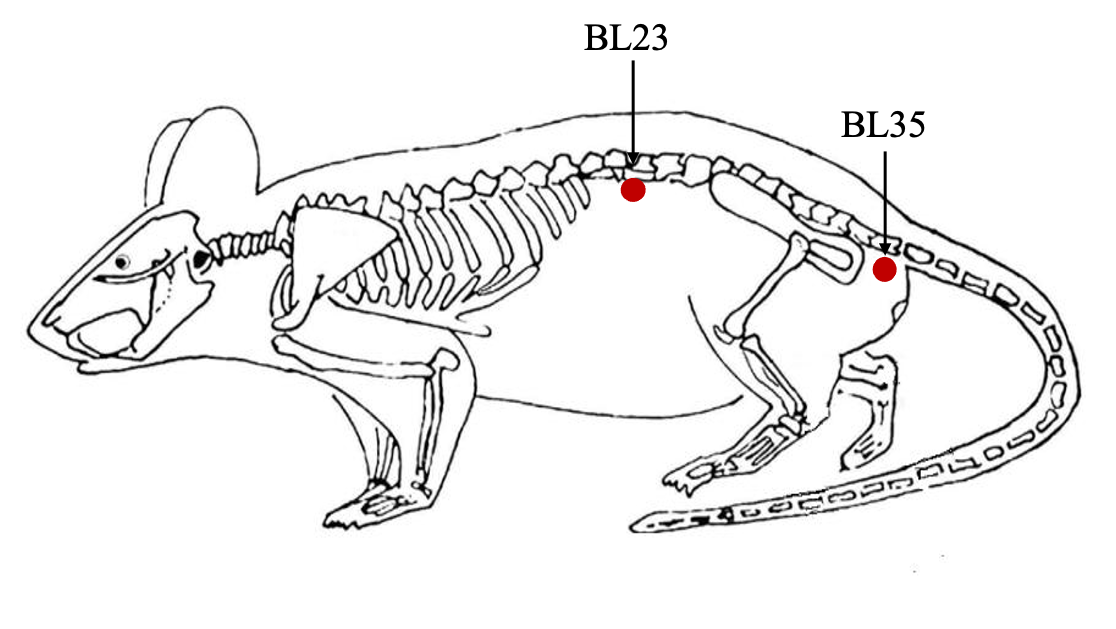

Supplement: Supplementary file 1 — (PNG 281 kb) [file 192_2022_5106_MOESM1_ESM.png]
